# Supplementary material for: Hippocampus mediates conceptual generalization of pain modulation
Source: bioRxiv. 2026 Oct 21:2026.10.16.759532. Preprint. [Version 1] doi: 10.64898/2026.10.16.759532 (PMC13738504; doi:10.64898/2026.10.16.759532)
Supplement: Supplementary file 1 [file NIHPP2026.10.16.759532v1-supplement-1.pdf]

bioRxiv preprint doi: https://doi.org/10.64898/2026.10.16.759532; this version posted October 21, 2026. The copyright holder for this preprint (which was not certified by peer review) is the author/funder, who has granted bioRxiv a license to display the preprint in perpetuity. It is made available under a CC-BY 4.0 International license.

Supplementary information:

Hippocampus mediates conceptual generalization of pain modulation

Dylan Sutterlin-Guindon[1,2], Tor D. Wager*[3], Leonie Koban*[45]

Affiliations.

1 Department of Psychology, University of Montreal, Canada

2 Centre de recherche de l'Institut universitaire de gériatrie de Montréal, Canada

3 Department of Psychological and Brain Sciences, Dartmouth College, Hanover, United States

4 Lyon Neuroscience Research Center (CRNL), CNRS, Inserm, Université Claude Bernard, Lyon 1, Bron, France

5 Le Vinatier Hospital, Bron, France

* shared senior authorship

Correspondence should be addressed to: Dr. Leonie Koban, SOCIALHEALTH team, Lyon Neuroscience Research Center (CRNL), Centre Hospitalier Le Vinatier, Bâtiment 462 Neurocampus Michel Jouvet, 95 boulevard Pinel, 69500 Bron; email: leonie.koban@cnrs.fr; phone: +33 (0)4 81 10 65 93

bioRxiv preprint doi: https://doi.org/10.64898/2026.10.16.759532; this version posted October 21, 2026. The copyright holder for this preprint (which was not certified by peer review) is the author/funder, who has granted bioRxiv a license to display the preprint in perpetuity. It is made available under a CC-BY 4.0 International license.

Supplementary Table 1. Path a effects from mediation analyses (FDR corrected, $q < 0.05$). This table reports positive and negative effects of the $GS_{HIGH\text{-}CAT} > GS_{LOW\text{-}CAT}$ contrast during medium-intensity heat stimulation. Brain regions are labeled using the combined anatomical atlas developed by the CANlab group, which integrates several parcellation schemes. Cortical regions (Ctx) are based on 468 parcels from the multimodal Glasser et al. (2016) atlas, basal ganglia subdivisions from Pauli et al. (2016, 2018), cerebellar regions from Diedrichsen et al. (2009), brainstem subdivisions from Fairbust et al. (2007) and from Shen et al. (2013). The full combined anatomical atlas can be downloaded from https://github.com/canlab/Neuroimaging_Pattern_Masks/tree/master/Atlases_and_parcellations/2018_Wager_combined_atlas, with additional documentation available at https://sites.google.com/dartmouth.edu/canlab-brainpatterns/brain-atlases-and-parcellations/2018-combined-atlas. In addition, large-scale functional networks are labeled based on the atlas by Schaefer et al. (2018).

Region	Volume (mm3)	X	Y	Z	maxZ	Large-scale region network
***Path a* positive effects ($GS_{HIGH\text{-}CAT} > GS_{LOW\text{-}CAT}$)**						
AMY L	3592	-22	-4	-20	3.94	Amygdala
AMY R	992	14	-2	-22	3.94	Amygdala
NAC R	128	6	2	-16	3.21	Basal ganglia
Bstem Ponscd	144	8	-36	-38	3.23	Brainstem
Bstem Pons R	144	12	-20	-36	3.3	Brainstem
Bstem Midb L	280	-8	-24	-18	3.45	Brainstem
Bstem Pons R	160	12	-16	-20	3.02	Brainstem
Cblm IX R	824	4	-44	-40	3.54	Cerebellum
Cblm VI R	1496	32	-44	-26	3.9	Cerebellum
Ctx a24 R	600	0	28	-4	3.74	Cortex Default Mode A
Ctx PHA3 L	400	-34	-34	-18	4.05	Cortex Default Mode C
Ctx PHA1 L	568	-26	-32	-12	3.46	Cortex Default Mode C
Ctx 6ma L	224	-16	-6	78	3.16	Cortex Fronto Parietal A
Ctx TGv L	272	-28	2	-48	3.89	Cortex Limbic
Ctx TGd R	1160	44	4	-38	4.11	Cortex Limbic
Ctx TGd R	128	54	6	-38	3.52	Cortex Limbic

bioRxiv preprint doi: https://doi.org/10.64898/2026.10.16.759532; this version posted October 21, 2026. The copyright holder for this preprint (which was not certified by peer review) is the author/funder, who has granted bioRxiv a license to display the preprint in perpetuity. It is made available under a CC-BY 4.0 International license.

Ctx TF R	424	40	-28	-20	3.42	Cortex Limbic
Ctx 6d L	1888	-24	-16	76	3.78	Cortex Somatomotor A
Ctx A5 R	160	58	-6	-2	3.07	Cortex Temporal Parietal
Ctx PoI2 L	1656	-40	-10	-6	4.19	Cortex Ventral Attention A
Ctx PF R	448	50	-30	30	3.6	Cortex Ventral Attention A

Path a **negative effects ($GS_{HIGH\text{-}CAT} > GS_{LOW\text{-}CAT}$)**

Ctx 9 46d R	192	26	44	36	-2.96	Cortex Ventral Attention B

bioRxiv preprint doi: https://doi.org/10.64898/2026.10.16.759532; this version posted October 21, 2026. The copyright holder for this preprint (which was not certified by peer review) is the author/funder, who has granted bioRxiv a license to display the preprint in perpetuity. It is made available under a CC-BY 4.0 International license.

Supplementary Table 2. Path b effects of mediation analyses (FDR corrected $q < 0.05$). Positive and negative effects of brain activity associated with pain ratings on a trial-by-trial basis, while controlling for generalization conditions ($GS_{HIGH\text{-}CAT}$ and $GS_{LOW\text{-}CAT}$).

Region	Volume (mm3)	X	Y	Z	maxZ	Large-scale region or network
***Path b* positive effects (increased activity predicts more pain, controlling for *path a*)**						
Multiple regions	75024	46	-10	6	5.19	Basal ganglia
Multiple regions	83688	-42	-10	10	4.97	Basal ganglia
Cau R	2520	14	26	2	3.89	Basal ganglia
Caudate Cp R	368	18	-4	24	3.46	Basal ganglia
Bstem Pons R	272	8	-18	-24	3.28	Brainstem
Bstem Midb L	280	-8	-24	-16	3.51	Brainstem
Bstem Midbd R	1408	14	-22	-6	3.86	Brainstem
Cblm VIIIa R	1128	28	-62	-48	3.75	Cerebellum
Cblm CrusII L	472	-32	-66	-46	4.18	Cerebellum
Cblm VIIIb L	280	-22	-46	-44	3.23	Cerebellum
Cblm V L	3216	-22	-36	-34	4.24	Cerebellum
Cblm CrusI R	1208	36	-74	-26	3.8	Cerebellum
Cblm CrusI R	160	26	-90	-26	3.17	Cerebellum
Ctx TGd L	744	-48	4	-38	4.38	Cortex Default Mode B
Ctx TGd L	160	-38	16	-40	3.43	Cortex Default Mode B
Ctx TGd L	152	-36	14	-36	3.23	Cortex Default Mode B
Ctx PGi L	2352	-44	-52	8	3.94	Cortex Default Mode B
Ctx MIP R	208	20	-58	36	3.3	Cortex Dorsal Attention A
Ctx IP2 L	792	-30	-56	34	3.82	Cortex Fronto Parietal A
Ctx 111 R	464	22	36	-10	4.01	Cortex Fronto Parietal B
Ctx PeEc R	504	18	0	-32	3.57	Cortex Limbic
Ctx OFC L	392	-8	16	-26	3.42	Cortex Limbic

bioRxiv preprint doi: https://doi.org/10.64898/2026.10.16.759532; this version posted October 21, 2026. The copyright holder for this preprint (which was not certified by peer review) is the author/funder, who has granted bioRxiv a license to display the preprint in perpetuity. It is made available under a CC-BY 4.0 International license.

Multiple regions	117240	2	-24	58	7.17	Cortex Somatomotor A
Ctx 4 R	6504	46	-10	42	4.37	Cortex Somatomotor A
Ctx RI L	608	-42	-40	14	3.41	Cortex Somatomotor B
Ctx 9 46d L	5776	-34	56	18	3.97	Cortex Ventral Attention B
Ctx a9 46v R	2672	38	48	18	3.68	Cortex Ventral Attention B
Ctx V2 R	616	26	-94	-16	3.75	Cortex Visual Central
Ctx LO1 R	872	36	-92	4	3.41	Cortex Visual Central
Ctx V2 R	176	18	-94	12	3.31	Cortex Visual Central
Multiple regions	144832	-4	-72	-2	5.3	Cortex Visual Peripheral
Ctx DVT L	192	-22	-68	20	2.98	Cortex Visual Peripheral
Thal VL	392	-14	-16	0	3.31	Diencephalon
Thal VA	144	-12	-6	6	3.03	Diencephalon
Thal Intralam	1176	2	-22	12	4.04	Diencephalon
No label	136	-48	-46	-6	3.68	No description
No label	288	-32	-44	6	3.71	No description
No label	224	-30	-50	14	3.1	No description
**Path b negative effects (reduced activity is associated with more pain, controlling for path a)**						
Ctx OFC L	128	-8	32	-30	-3	Cortex Limbic

bioRxiv preprint doi: https://doi.org/10.64898/2026.10.16.759532; this version posted October 21, 2026. The copyright holder for this preprint (which was not certified by peer review) is the author/funder, who has granted bioRxiv a license to display the preprint in perpetuity. It is made available under a CC-BY 4.0 International license.

Supplementary Table 3. *Path ab* effects of mediation analyses (FDR corrected $q < 0.05$). Positive mediation effects indicate that increased pain-evoked activity during generalization ($GS_{HIGH\text{-}CAT} > GS_{LOW\text{-}CAT}$) was associated with more pain ratings.

Region	Volume (mm3)	X	Y	Z	maxZ	Large-scale region or network
***Path ab* positive effects (mediation of conceptual generalization effect on pain ratings)**						
Multiple regions	9376	2	4	-6	5.64	Basal ganglia
V Striatum R	624	16	22	-4	4.71	Basal ganglia
Cau R	616	24	-32	14	5.03	Basal ganglia
Caudate Ca R	160	20	20	6	3.91	Basal ganglia
Caudate Cp L	496	-8	12	16	6.35	Basal ganglia
Caudate Cp L	248	-12	18	14	4.17	Basal ganglia
Bstem Med R	320	2	-38	-46	3.62	Brainstem
Bstem Ponscv R	464	2	-24	-46	4.06	Brainstem
Multiple regions	11488	-14	-30	-6	8.04	Brainstem
Cblm CrusII R	864	14	-84	-40	4.26	Cerebellum
Cblm CrusI R	264	44	-50	-36	3.75	Cerebellum
Cblm CrusII R	744	10	-88	-32	5.15	Cerebellum
Cblm CrusI R	408	44	-78	-30	3.97	Cerebellum
Cblm VI R	1808	34	-46	-24	5.04	Cerebellum
Cblm CrusI L	736	-44	-40	-28	4.03	Cerebellum
Cblm CrusII R	568	16	-90	-26	4.06	Cerebellum
Cblm I IV R	208	10	-34	-14	3.59	Cerebellum
Ctx TE1a R	2008	60	-14	-16	5.1	Cortex Default Mode A
Ctx 10d L	192	-20	66	14	3.04	Cortex Default Mode A
Ctx 31pd R	200	10	-50	26	3.24	Cortex Default Mode A
Ctx RSC R	672	6	-32	26	4.22	Cortex Default Mode A
Ctx 7m L	1464	-8	-56	30	3.56	Cortex Default Mode A

bioRxiv preprint doi: https://doi.org/10.64898/2026.10.16.759532; this version posted October 21, 2026. The copyright holder for this preprint (which was not certified by peer review) is the author/funder, who has granted bioRxiv a license to display the preprint in perpetuity. It is made available under a CC-BY 4.0 International license.

Ctx PGs R	2200	40	-70	38	4.61	Cortex Default Mode A
Ctx 8Ad L	248	-20	32	36	3.82	Cortex Default Mode A
Ctx 8Ad R	392	26	22	40	4.63	Cortex Default Mode A
Ctx PGs L	1064	-44	-68	42	4.15	Cortex Default Mode A
Ctx 8Ad R	272	14	34	42	3.96	Cortex Default Mode A
Ctx TGd L	6384	-40	8	-28	7.92	Corte Default Mode B
Ctx TE1a_L	800	-64	-16	-28	5.29	Cortex Default Mode B
Ctx 47s L	128	-30	18	-26	3.33	Cortex Default Mode B
Ctx 45 L	568	-58	30	16	3.9	Cortex Default Mode B
Ctx POS1 R	192	8	-52	14	3.06	Cortex Default Mode C
Ctx PGi R	160	40	-58	28	3.36	Cortex Default Mode C
Ctx RSC L	824	-6	-16	30	5.62	Cortex Default Mode C
Ctx TE2p L	344	-48	-24	-26	3.87	Cortex Dorsal Attention A
Ctx FFC L	400	-50	-60	-16	3.73	Cortex Dorsal Attention A
Ctx TE1m L	176	-54	-28	-18	3.9	Cortex Fronto Parietal B
Ctx a10p R	328	22	62	-10	3.65	Cortex Fronto Parietal B
Ctx a47r R	576	30	62	2	3.96	Cortex Fronto Parietal B
Ctx POS2 R	440	0	-70	26	3.2	Cortex Fronto Parietal C
Ctx TGv R	1016	42	-6	-42	4.01	Cortex Limbic
Ctx TGd R	472	24	16	-40	4.88	Cortex Limbic
Ctx TE2a L	184	-52	-26	-28	3.42	Cortex Limbic
Ctx TGd R	528	42	6	-22	5.68	Cortex Limbic
Ctx 13l L	288	-32	32	-20	3.88	Cortex Limbic
Ctx TF R	192	38	-26	-14	3.37	Cortex Limbic
Ctx 10pp R	640	18	56	-10	4.17	Cortex Limbic
Ctx 3a L	960	-50	-14	24	4.56	Cortex Somatomotor A
Ctx 4 L	152	-50	-6	24	3.33	Cortex Somatomotor A

bioRxiv preprint doi: https://doi.org/10.64898/2026.10.16.759532; this version posted October 21, 2026. The copyright holder for this preprint (which was not certified by peer review) is the author/funder, who has granted bioRxiv a license to display the preprint in perpetuity. It is made available under a CC-BY 4.0 International license.

Ctx 1 L	992	-58	-24	50	4.14	Cortex Somatomotor A
Ctx 3b R	152	46	-14	42	3.48	Cortex Somatomotor A
Ctx 6mp L	536	-16	-22	64	3.83	Cortex Somatomotor A
Ctx 6v L	280	-64	6	28	5.18	Cortex Somatomotor B
Ctx STGa R	432	52	8	-22	3.32	Cortex Temporal Parietal
Ctx A5 L	2480	-64	-16	-8	6.98	Cortex Temporal Parietal
Ctx A5 L	200	-62	0	-10	4.98	Cortex Temporal Parietal
Ctx STSdp R	1144	44	-28	4	4.08	Cortex Temporal Parietal
Ctx AAIC R	1992	26	14	-18	3.8	Cortex Ventral Attention A
Ctx 6r L	320	-62	12	20	4.59	Cortex Ventral Attention A
Ctx SCEF L	472	-4	-2	50	3.63	Cortex Ventral Attention A
Ctx SCEF R	392	4	8	52	3.8	Cortex Ventral Attention A
Ctx AVI L	320	-30	18	-8	3.51	Cortex Ventral Attention B
Ctx AVI L	864	-30	26	-8	3.93	Cortex Ventral Attention B
Ctx FOP5 R	384	38	30	-4	3.71	Cortex Ventral Attention B
Ctx 46 L	568	-50	40	16	3.55	Cortex Ventral Attention B
Ctx 9 46d L	488	-28	38	22	3.95	Cortex Ventral Attention B
Ctx 46 R	280	42	50	26	3.65	Cortex Ventral Attention B
Ctx PIT L	560	-52	-72	-10	3.71	Cortex Visual Central
Ctx V1 L	1048	-4	-58	4	3.75	Cortex Visual Peripheral
Ctx ProS L	552	-28	-54	4	3.47	Cortex Visual Peripheral
Ctx V6A R	320	20	-86	42	3.26	Cortex Visual Peripheral
Thal Pulv	5080	10	-30	2	6.78	Diencephalon
No label	264	34	-46	0	5.4	No description
No label	352	32	-50	6	4.07	No description
No label	624	26	-46	20	4.54	No description
No label	224	4	8	26	4.33	No description

bioRxiv preprint doi: https://doi.org/10.64898/2026.10.16.759532; this version posted October 21, 2026. The copyright holder for this preprint (which was not certified by peer review) is the author/funder, who has granted bioRxiv a license to display the preprint in perpetuity. It is made available under a CC-BY 4.0 International license.

Supplementary Table 4. Moderation of *Path ab* effects by individual differences in explicit learning (FDR corrected $q < 0.05$).

**Region**	**Volume (mm3)**	**X**	**Y**	**Z**	**maxZ**	**Large-scale region or network**
**Moderation of *path ab* effects by individual differences in explicit learning**						
CA3 Hippocampus	304	-32	-22	-12	3.62	Hippocampus

bioRxiv preprint doi: https://doi.org/10.64898/2026.10.16.759532; this version posted October 21, 2026. The copyright holder for this preprint (which was not certified by peer review) is the author/funder, who has granted bioRxiv a license to display the preprint in perpetuity. It is made available under a CC-BY 4.0 International license.

Supplementary References

Diedrichsen, J., Balsters, J. H., Flavell, J., Cussans, E., & Ramnani, N. (2009). A Probabilistic MR Atlas of the Human Cerebellum. *NeuroImage.* 46(1):39-46.

Fairhurst, M., Wiech, K., Dunckley, P. & Tracey, I. (2007). Anticipatory Brainstem Activity Predicts Neural Processing of Pain in Humans. *Pain.* 128:101-10.

Glasser, M. F., Coalson, T. S., Robbinson, E. C., Hacher, C. D, Harwell, J., Yacoub, E., Ugurnil, K., Andersson, J., Beckmann, C. F., Jenkinson, M., Smith, S. M. & Van Essen, D. C. (2016). A Multi-Modal Parcellation of Human Cerebral Cortex. *Nature*. 536:171-78.

Pauli, W. M., Nili, A. N., & Tyszka, J. M (2018). A High-Resolution Probabilistic in Vivo Atlas of Human Subcortical Brain Nuclei. *Scientific Data.* 5:180063.

Pauli, W. M., O'Reilly, R. C., Yarkoni, T. & Wager, T. D. (2016). Regional Specialization within the Human Striatum for Diverse Psychological Functions. *PNAS*. 113(7):1907-12.

Schaefer A, Kong R, Gordon EM, Laumann TO, Zuo XN, Holmes AJ, Eickhoff SB, Yeo BTT. Local-Global Parcellation of the Human Cerebral Cortex from Intrinsic Functional Connectivity MRI. Cereb Cortex. 2018 Sep 1;28(9):3095-3114.

Shen, X., Tokoglu, F., Papademetris, X. & Constable, R. T. (2013). Groupwise Whole-Brain Parcellation from Resting-State fMRI Data for Network Node Identification. *NeuroImage*. 82:403–15.
